# Decreased RIG-I expression is associated with poor prognosis and promotes cell invasion in human gastric cancer

**DOI:** 10.1186/s12935-018-0639-3

**Published:** 2018-09-19

**Authors:** Lujun Chen, Jun Feng, Shaoxian Wu, Bin Xu, You Zhou, Changping Wu, Jingting Jiang

**Affiliations:** 1 Department of Tumor Biological Treatment, The Third Affiliated Hospital of Soochow University, Changzhou, 213003 Jiangsu China; 2 Jiangsu Engineering Research Center for Tumor Immunotherapy, The Third Affiliated Hospital of Soochow University, Changzhou, 213003 Jiangsu China; 3 Institute of Cell Therapy, The Third Affiliated Hospital of Soochow University, Changzhou, 213003 Jiangsu China

**Keywords:** RIG-I, Gastric cancer, Immunohistochemistry, RNAi, Prognosis

## Abstract

**Background:**

Retinoic acid-induced protein I (RIG-I), known as a cytoplastic pattern recognition receptor, can recognize exogenous viral RNAs, and then initiate immune response. Recently, numerous studies also showed that RIG-I play an important role in oncogenesis and cancer progression as well. As of now, the expression pattern and the role of RIG-I in gastric cancer still remain largely unexplored. In this study, we investigated the clinical associations of RIG-I expression in human gastric cancer tissues and further explore its important contribution in the regulation of malignant phenotype of gastric cancer cells.

**Methods:**

Immunohistochemistry was performed to study the correlation between patients’ clinical parameters and RIG-I expression in gastric cancer tissues. Knockdown of RIG-I was achieved by RNAi technology to examine the contribution of RIG-I in the regulation of biological functions in the cell lines of human gastric cancer. The Affymetrix GeneChip was performed to figure out the differential gene expression profile between RIG-I wild type and RIG-I knockdown cell lines of gastric cancer.

**Results:**

Immunohistochemistry result demonstrated that the expression of RIG-I in gastric cancer tissues significantly correlated with pathological stage and patients’ prognoses. Furthermore, decreased RIG-I expression in human gastric cancer cell lines could significantly increase the cell migration, cell viability, and the ratio of cells in G2/M phase. Our microarray analysis also revealed that the differentially expressed gene profiles were enriched in related signal pathways or biological processes in KEGG or GO analysis respectively.

**Conclusions:**

Our present findings showed that the decreased RIG-I expression significantly correlated with patients’ prognoses, and such down-regulation could promote the cell invasion in this malignancy.

**Electronic supplementary material:**

The online version of this article (10.1186/s12935-018-0639-3) contains supplementary material, which is available to authorized users.

## Background

Chronic inflammation is greatly related to the increasing risk of many human cancers [1]. For example, *Helicobacter pylori* (HP), has been widely accepted as one of the most important reasons to induce the infection of gastric mucosa, and then lead to acute inflammation, chronic inflammation, gastric atrophy, intestinal metaplasia, dysplasia, and finally gastric cancer [2, 3].

As we know, the pattern recognition receptors (PRRs), those could be expressed on both non-immune and innate immune cells, have been found to role importantly in detecting foreign pathogens and then initiating innate or adaptive immune response. PRRs can be activated by specific pathogen-associated molecular patterns (PAMPs) in microbial and/or danger-associated molecular patterns (DAMPs) on the surface or released by damaged cells [4]. There are several subgroups of PRRs. Based on their localization in cells, they can be classified as the membrane-associated toll-like receptors, C-type lectin receptors, and the cytosolic nod-like receptors, retinoic acid-induced protein I like receptors, AIM2-like receptors [4, 5].

As an important member of the RLR group, RIG-I, also known as DDX58, is induced by all-trans-retinoic acid to regulate the differentiation of granulocytes from APL cells [6]. RIG-I contains two N-terminal caspase recruitment domains, a central DExH box helicase/ATPase and a C-terminal regulatory domain, and the N-terminal caspase recruitment domains could directly induce type I interferon expression and tumor cell apoptosis [7]. RIG-I was found to be served as a cytoplastic PRR to initiate the innate antiviral immunity by recognizing exogenous viral RNAs. However, recent studies have shown that RIG-I could also sense endogenous RNAs to participate in particular cellular process under some circumstances [6]. Zhu et al. have shown that the expression of RIG-I is decreased in colorectal cancer tissues in contrast to the adjacent normal tissues, and RIG-I knock-out mice are more susceptible to colitis-associated cancer [8].

Herein, we aimed to investigate the association between the RIG-I expression and the clinical parameters and outcomes of gastric cancer patients. Furthermore, we also evaluated the regulatory role of RIG-I in gastric cancer on cellular level. Our immunohistochemistry results demonstrated that the RIG-I expression level is positively correlated with the survival of gastric cancer patients. And reduced RIG-I expression significantly increased the cell abilities such as migration, proliferation and invasion, in cell lines of gastric cancer. Thus, our results indicated that the decreased RIG-I expression was positively correlated with poor prognosis, and such down-regulation significantly promoted the cell malignancy in human gastric cancer.

## Materials and methods

### Patients and tissue samples

The gastric cancer tissue array (Catalog number: HStm-Ade180Sur-05) from Shanghai Outdo Biotech Co., Ltd. (Shanghai, P. R. China) has been used in our previous study [9]. Table 1 presents the detailed clinical parameters of the patients. The survival data of all the patients were collected. The incomplete tissue points and several missing tissue points were excluded when performing the heat-induced antigen retrieval. Therefore, a total of 84 cases were finally included in the statistical analysis. The protocols for the present study were approved by the ethics committee of our hospital.

**Table 1:** Correlation between RIG-I expression in gastric cancer tissues and patients’ clinical parameters

Clinical parameters	Cases	RIG-I expression level		*χ* ^2^	*P*
		High (H-score ≥ 45)	Low (H-score < 45)		
Gender				0.243	0.622
Male	53	38	15		
Female	30	23	7		
Age (years)				0.377	0.539
≥ 60	56	40	16		
< 60	27	21	6		
Tumor size (cm)				0.683	0.409
≥ 5	41	31	10		
< 5	41	34	7		
Pathological stage				4.668	*0.031*
I + II	17	16	1		
III + IV	66	45	21		
Tumor stage				0.5901	0.442
T1 + T2	12	10	2		
T3 + T4	70	51	19		
Lymph node metastasis				0.326	0.568
Yes	65	49	16		
No	19	17	2		
Distant metastasis				0.881	0.348
Yes	10	9	1		
No	74	57	17		
TNM stage				0.561	0.454
I + II	33	26	7		
III + IV	49	35	14		

### Antibodies and other reagents

Rabbit against human RIG-I (AB45428, Abcam, Cambridge, MA, USA), HRP-conjugated secondary antibodies (K500711, Glostrup, Denmark), rabbit anti-human GAPDH antibody (Sigma, St. Louis, MO, USA), RNeasy Mini Kit (Qiagen, Valencia, CA, USA), SYBR Green Master Mix kit (Takara, Dalian, China), fetal bovine serum (Gibco, Cambrex, MD, USA), RPMI-1640 medium (Gibco, Cambrex, MD, USA) were used in the present study.

### Immunohistochemistry and the evaluation of immunostaining

Immunohistochemical staining was performed using the Envision™ method as described in our previous study [10]. In brief, the 3-μm sections from the gastric cancer tissue array block was prepared, and then dewaxed by using xylene and rehydrated in a graded series of alcohols. Then, the antigen retrieval was conducted by heating the tissue section at 100 °C for 30 min in an EDTA solution (1 mM, pH 9.0). Cooled tissue sections were immersed in 0.3% hydrogen peroxide solution for 15 min to block endogenous peroxidase activity, rinsed with PBS for 5 min and blocked with 3% BSA solution at room temperature for 30 min. Next, the slides were incubated with anti-human RIG-I (1:800) at 4 °C overnight and then incubated with HRP-conjugated secondary antibody. The evaluation of immunohistochemical staining was performed by using *H*-*score* method as reported in our previous studies [9–13].

### shRNA

The human gastric cancer cell lines SGC-7901 and AGS cells were selected and used for the knockdown of RIG-I expression. Small hairpin RNA (shRNA) against the human RIG-I gene (NM_014314.2; GenBank) was obtained from Shanghai Generay Biotech Co., Ltd. (Shanghai, China) and cloned into a lentiviral vector pLKO.1-GFP encoding green fluorescent protein (GFP). The shRNA target sequence against RIG-I was as follows: 5′-CCAGAATTATCCCAACCGAT-3′. The recombinant RIG-I-targeting lentivirus (LV-RIG-I-shRNA virus) and control mock lentivirus (LV-NC virus) were prepared and transfected into SGC-7901 and AGS cells respectively. The infected cells were analyzed by flow cytometry (Canto II, BD, USA), and the GFP-positive cells from the two groups were subsequently sorted using an Aria II flow sorter (BD Bioscience, NJ, USA) and then named as LV-RIG-I-shRNA or LV-NC, respectively.

### Cell culture and treatment

All cells were cultured in RPMI-1640 medium supplemented with 10% fetal bovine serum. Lentivirus was used to establish individual stable cells. The cell viability assay was performed using Cell Counting Kit-8 (Catalog Number: C0038, CCK-8, Beyotime), the cell migration ability was assessed by wound healing assay, and the cell invasion ability was evaluated by standard 24-well Boyden invasion chambers (Catalog number: 353097, BD Biosciences). Cell cycle assay was performed by flow cytometry using the cell cycle kit (Catalog Number: 81845, Sigma).

### Real-time polymerase chain reaction

The real-time polymerase chain reaction was performed and the housekeeping gene GAPDH was used as described in our previous study [9]. The sequences of the primers for RIG-I were listed as follows, forward primer: 5´-AGAGCACTTGTGGACGCTTT-3´, RIG-I reverse primer: 5´-TCAGCAACTGAGGTGGCAAT-3´. The relative expression level of RIG-I was calculated using the 2^−∆∆CT^ method.

### Western blotting analysis

Western blotting analysis was performed as described in our previous study [10].

### Agilent lncRNA microrray analysis

The Agilent Human lncRNA Microrrays (V6) were performed on the Affymetrix Microarray-Based Gene Expression Analysis platform by Oebiotech Co. (Shanghai, P. R. China) as described previously [14]. Differentially expressed genes or lncRNAs were then identified through fold change. The threshold set for up- and down-regulated genes was a fold change ≥ 2.0. Afterwards, GO analysis and KEGG analysis were applied to determine the roles of these differentially expressed mRNAs.

### Statistical analyses

The paired Student’s *t*-test, the Wilcoxon signed-rank test, the Chi square test or the Log-rank survival analysis was used where appropriate. All the statistical analyses were performed using the GraphPad Prism 5.0. *P *< 0.05 was deemed as statistically significant.

## Results

### Expression of RIG-I in adjacent normal tissues and gastric cancer tissues

**Fig. 1:**
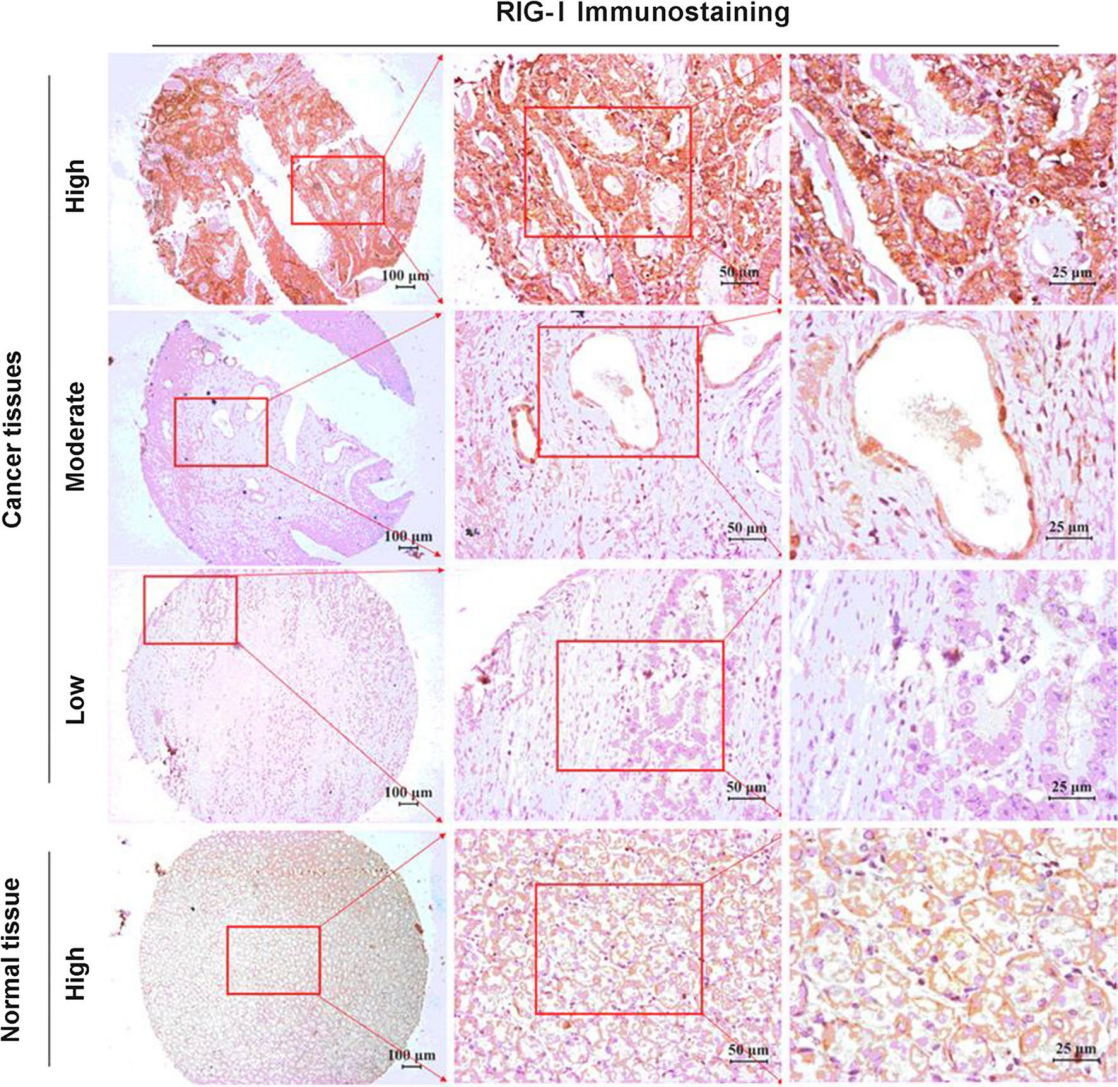
RIG-I Immunostaining in gastric tissues. RIG-I staining could be found in the cytoplasm of gastric cancer cells and gastric mucosal epithelial cells. Higher RIG-I expression could be found in normal gastric tissues as well as highly differentiated gastric cancer tissues, and lower RIG-I expression was found in moderately and poorly differentiated gastric cancer tissues

**Fig. 2:**
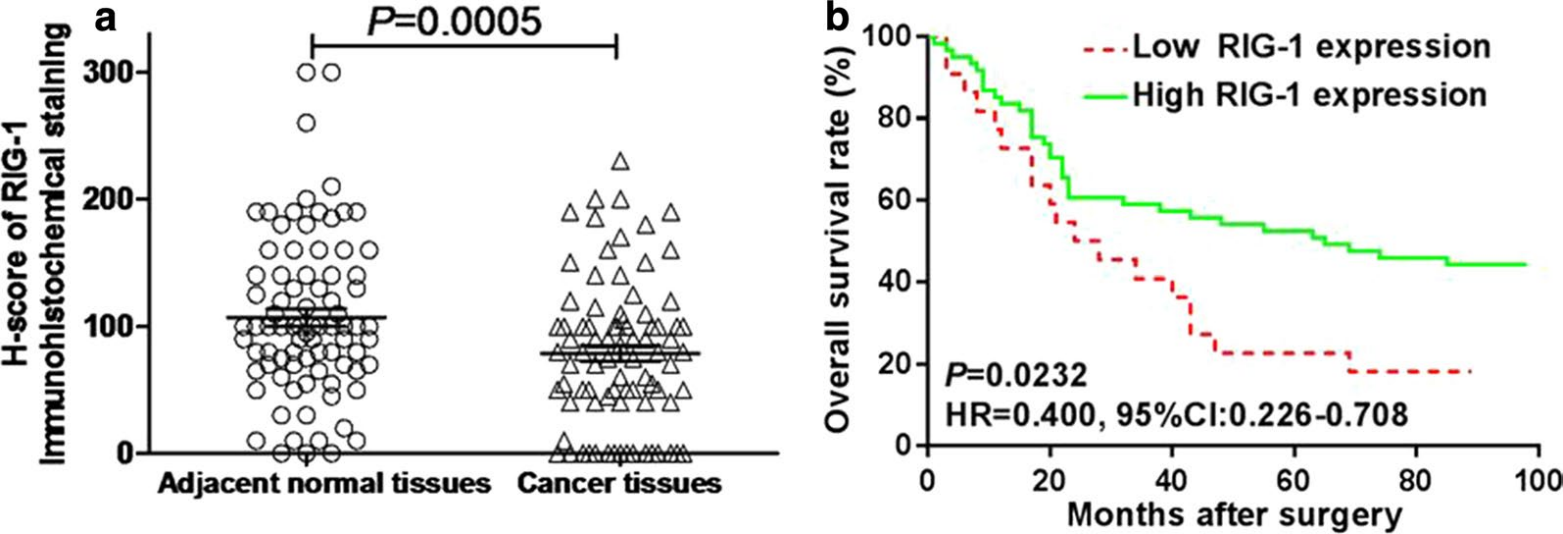
RIG-I expression in gastric cancer tissues and its prognostic value. **a** The immunostaining intensity of RIG-I in gastric cancer tissues was significantly lower than that in adjacent normal gastric cancer tissues (*U *= 1490, *P *= 0.0005). **b** The Log-rank survival analysis showed that the overall survival of the patients with higher RIG-I expression was significantly better than those with lower RIG-I expression (*P *= 0.0232, HR = 0.400, 95% CI 0.226–0.708)

To evaluate the clinical relevance of RIG-I in gastric cancer, immunohistochemical assay was performed with specific antibody against RIG-I of the gastric cancer tissue assay composed of 90 patient specimens. The RIG-I positive immunostaining revealed that RIG-I was predominately localized in the cytoplasm both in tumor cells and normal mucosal epithelial cells (Fig. 1). *H*-*score* evaluation of gastric specimens showed a significantly lower RIG-I expression level in tumors than in adjacent normal tissues with the median *H*-*scores* are 80 (0–230) and 100 (0–300) respectively (*U *= 1490, *P *= 0.0005, Fig. 2a).

### Correlation between RIG-I expression in gastric cancer tissues and clinical parameters as well as patients’ prognoses

**Fig. 3:**
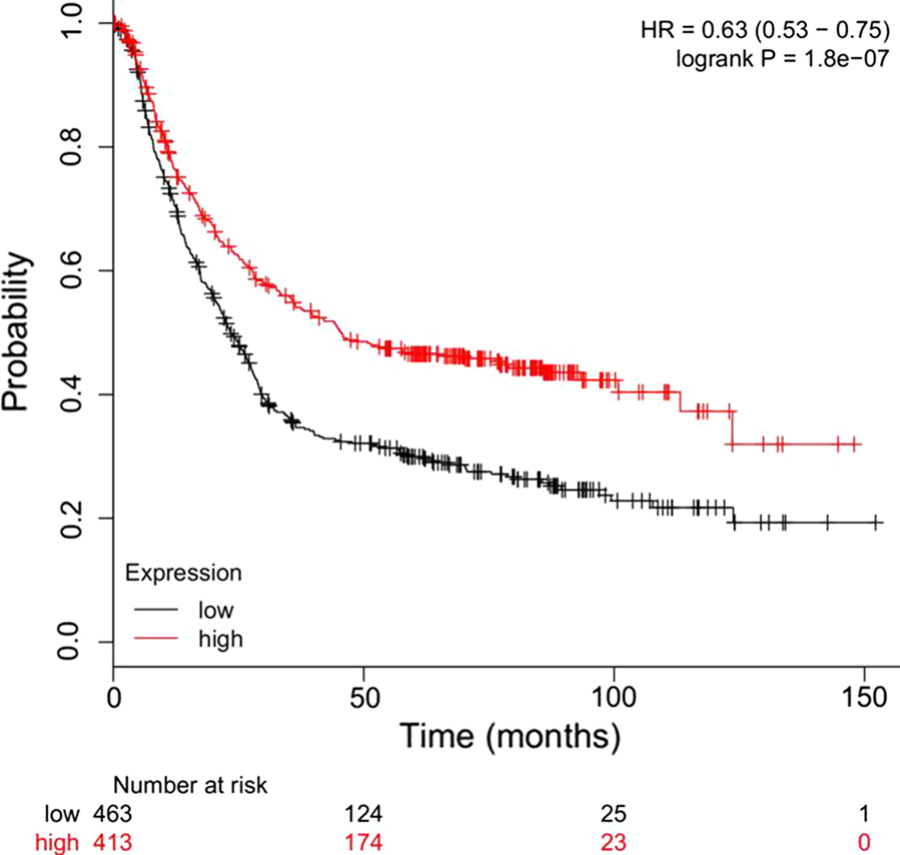
Prognostic value of the RIG-I expression at the mRNA level through the data of KMplot database. The prognostic value of RIG-I expression at the mRNA level was verified according to the data of KMplot database (http://kmplot.com/analysis/), and results indicated that the higher expression of RIG-I at the mRNA level was significantly associated with a better survival of the gastric cancer patients (*P *< 0.0001, HR = 0.63, 95% CI 0.53–0.75)

To further investigate the clinical relevance of RIG-I in human gastric cancer, we summarized several parameters of the patients in the human gastric cancer tissue assay and analyzed the correlation of RIG-I expression with these parameters. Results showed that the RIG-I expression level was only correlated with the pathological stage of the patients (*χ*^2^ = 4.668, *P *= 0.031), and there was no correlation with other parameters such as gender, age, tumor size, tumor stage, lymph node metastasis or TNM stage, as indicated in Table 1. Furthermore, the patients with higher RIG-I expression level (*H*-*score *> 45) exhibited a higher overall survival rate than those with lower RIG-I expression (*H*-*score *≤ 45) (*P *= 0.0232; HR = 0.400; 95% CI 0.226–0.708, Fig. 2b). Moreover, we also verified the prognostic value of RIG-I mRNA expression level according to the KMplot database (http://kmplot.com/analysis/), and the results indicated that the higher expression of RIG-I was significantly associated with a better survival of the gastric cancer patients (*P *< 0.0001, HR = 0.63, 95% CI 0.53–0.75, Fig. 3). The COX model revealed that the RIG-I expression level and the TNM stage could function as independent prognostic predictors in human gastric cancer (*P *= 0.007 and *P *= 0.039, respectively, Table 2).

**Table 2:** Cox model analysis for the correlation between RIG-I expression and patients’ clinical parameters

Clinical parameters	Uni-variate		Multi-variate	
	HR (95% CI)	*P*	HR (95% CI)	*P*
Gender (M/F)	0.924 (0.528–1.616)	0.781	0.733 (0.399–1.350)	0.319
Age (years) (≥ 60/< 60)	1.391 (0.736–2.536)	0.281	1.916 (1.010–3.636)	*0.047*
Tumor size (≥ 5 cm/< 5 cm)	1.726 (0.999–2.984)	0.051	1.077 (0.575–2.017)	0.816
Pathological stage (III + IV/I + II)	1.961 (0.883–4.355)	0.098	0.860 (0.358–2.067)	0.860
Tumor stage (T3 + T4/T1 + T2)	2.564 (0.923–7.127)	0.071	1.705 (0.588–4.941)	0.326
Lymph node metastasis (yes/no)	2.494 (1.170–5.314)	*0.018*	1.837 (0.621–5.437)	0.272
Distant metastasis (yes/no)	2.547 (1.187–5.466)	*0.016*	2.117 (0.907–4.940)	0.083
TNM stage (III + IV/I + II)	3.017 (1.620–5.617)	*0.000*	2.503 (1.047–5.980)	*0.039*
RIG-I expression (High/low)	0.400 (0.226–0.708)	*0.023*	0.404 (0.210–0.777)	*0.007*

### Knockdown expression of RIG-I in SGC-7901 and AGS cell lines

**Fig. 4:**
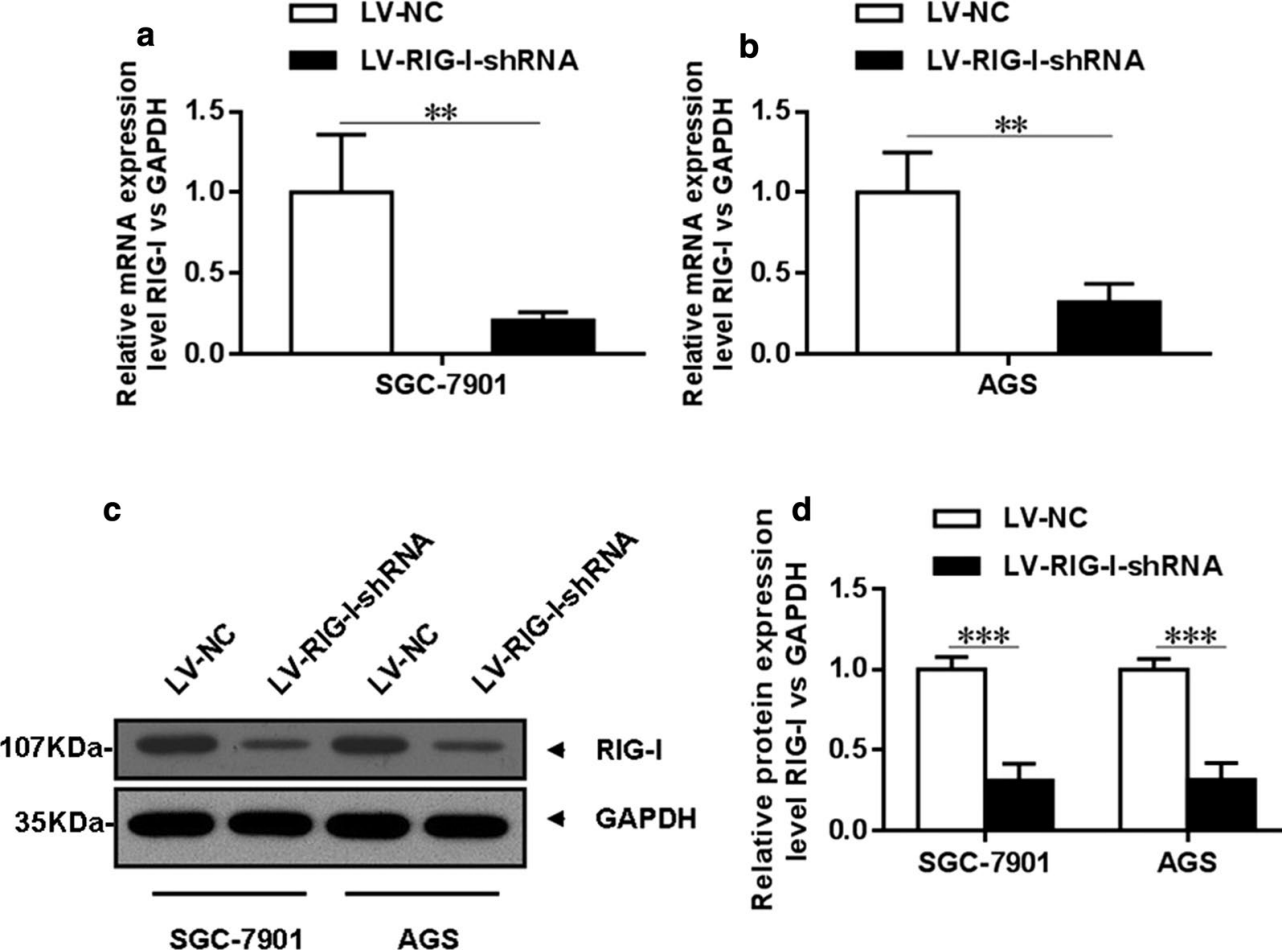
Knockdown of RIG-I in human gastric cancer cell lines. **a**, **b** The RIG-I expression at the mRNA level was significantly decreased after knockdown by using RNAi in SGC-7901 and AGS cells (both *P *< 0.01). **c**, **d** Western blotting analysis showed that the RIG-I expression at the protein level was significantly decreased after knockdown by using RNAi in SGC-7901 and AGS cells (both *P *< 0.001)

Figure 4a, b showed that after knockdown of RIG-I expression in SGC-7901 and AGS, the mRNA level was significantly decreased in those two cell lines (both *P *< 0.005). Moreover, Western blotting analysis showed that the RIG-I expression at the protein level, the RIG-I expression was significantly decreased after knockdown in SGC-7901 as well as AGS cells (both *P *< 0.001) (Fig. 4c, d).

### Decreased RIG-I in human gastric cancer cells could regulate the cellular functions

**Fig. 5:**
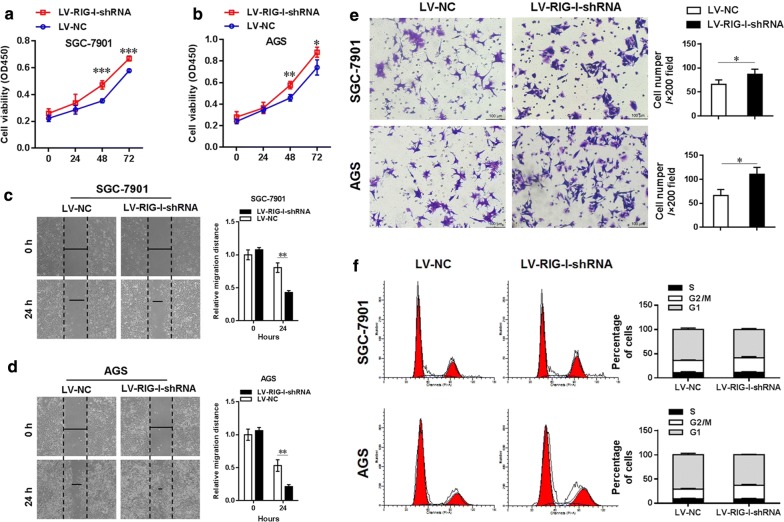
Effect of knockdown of RIG-I on cellular functions of gastric cancer cell lines. **a** Knockdown of RIG-I in SGC-7901 cells could increase the cell viability at 48 h and 72 h post-transfection (both *P *< 0.001). **b** Knockdown of RIG-I in AGS cells could increase the cell viability at 48 h and 72 h post-transfection (*P *< 0.01 and *P *< 0.05, respectively). **c** Knockdown of RIG-I significantly increased the cell migration ability in SGC-7901 cells at 24 h after scraping (*P *< 0.01). **d** Knockdown of RIG-I could increase the cell migration ability in AGS cells at 24 h after scraping (*P *< 0.01). **e** The number of crystal violet-stained cells was significantly increased after RIG-I knockdown expression in both two cell lines (*P *< 0.05), respectively. **f** The ratio of gastric cells in G2/M phase was significantly increased in after RIG-I knockdown expression in both SGC-7901 and AGS cells (*P *< 0.05 and *P *< 0.01, respectively)

**Fig. 6:**
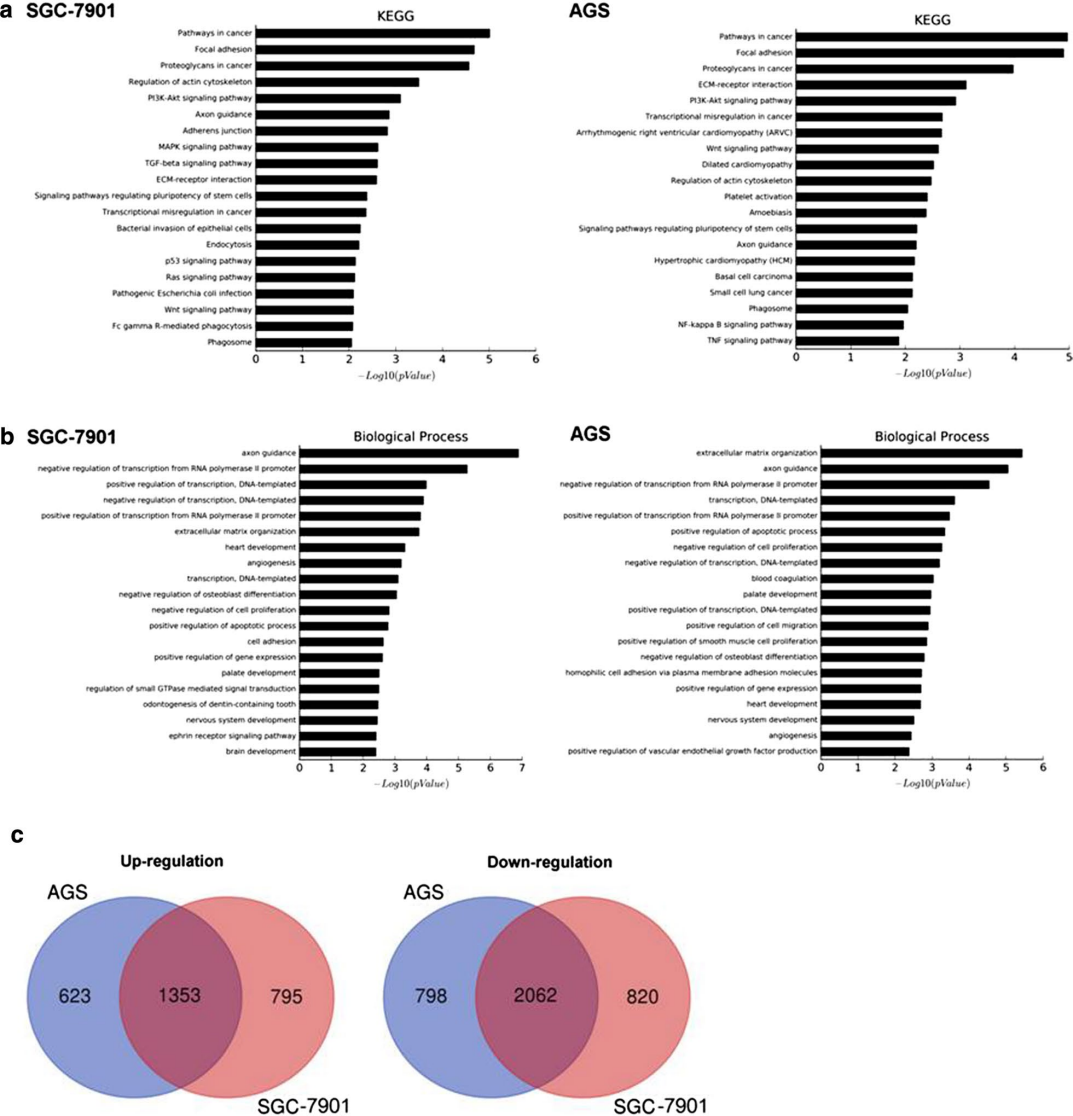
Microarray analysis of the differentially expressed gene profiles of RIG-I knockdown in gastric cancer cell lines. The Affymetrix GeneChip was used to investigate the differential gene expression profile of RIG-I knockdown in gastric cancer cell lines AGS and SGC-7901. **a** KEGG analysis showed top 20 signal pathways of differentially expressed gene profiles in SGC-7901 and AGS cell lines. **b** GO analysis showed top 20 biological process GO terms of differentially expressed gene profiles in SGC-7901 and AGS cell lines. **c** Venn diagram showed the overlap of differentially expressed genes in SGC-7901 and AGS cell lines (left, up-regulated genes; right, down-regulated genes)

**Fig. 7:**
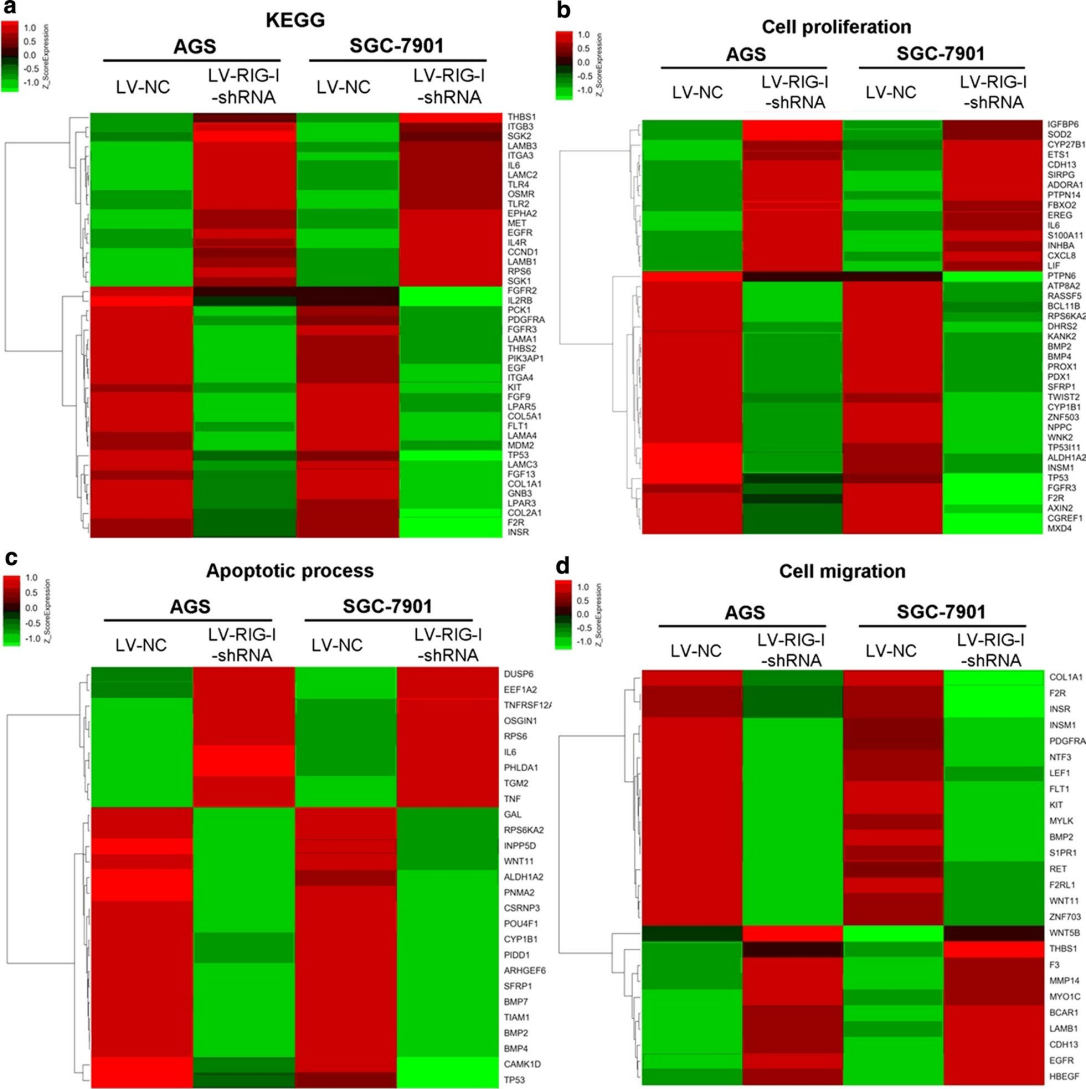
Heatmap of significantly dysregulated genes enriched in PI3K-Akt pathway as well as several biological processes. We select the absolute Fold change ≥ 10.0 as cut off, and the enriched significantly dysregulated genes were involved in the Heatmap analysis. **a** Enriched significantly dysregulated genes from KEGG analysis of PI3K-Akt signal pathway. **b** Enriched significantly dysregulated genes from GO analysis of cell proliferation. **c** Enriched significantly dysregulated genes from GO analysis of cell apoptosis. **d** Enriched significantly dysregulated genes from GO analysis of cell migration

Figure 5a, b show that RIG-I knockdown expression in SGC-7901 and AGS significantly increased the cell viability at both 48 h and 72 h after transfection (in SGC7901 cells, both *P *< 0.001; in AGS cells, *P *< 0.01 and *P *< 0.05, respectively). Figure 5c, d showed that after knockdown of RIG-I in both SGC-7901 and AGS cells, the migrated distance in the LV-RIG-I-shRNA group was significantly increased in contrast to the LV-NC group at 24 h after cell scraping (*P *< 0.01, respectively). Moreover, the transwell migration assay was performed to evaluate the cell migration ability of SGC-7901 and AGS cells between the LV-RIG-I-shRNA group and LV-NC group. Figure 5e showed that the number of crystal violet-stained cells significantly increased after RIG-I knockdown expression in both SGC-7901 and AGS cells (both *P *< 0.05), and the cell cycle assay also revealed that the ratio of gastric cells in G2/M phase significantly increased after RIG-I knockdown expression in those two cell lines (*P *< 0.05 and *P *< 0.01, respectively, Fig. 5f). Moreover, our Microarray analysis (data are shown in Additional files 1 and 2) also show that the differentially expressed gene profiles also enriched in the signal pathways such as PI3K/Akt in KEGG analysis (Fig. 6a), and in the biological processes such as cell proliferation and migration in GO analysis (Fig. 6b). The Venn diagram also showed that there was a big overlap of differentially expressed genes in SGC-7901 and AGS cell lines toward RIG-I knockdown expression (Fig. 6c). Heatmap of significantly dysregulated genes enriched in PI3K-Akt pathway as well as several biological processes was also shown in Fig. 7.

## Discussion

RIG-I can not only induce type I IFN in response to different RNA viruses, but also could be involved in the progression of many human cancers. Thus, RIG-I has potential implications for therapeutic strategy against human cancers [6]. RIG-I was first identified to be induced by all-trans-retinoic acid to regulate the differentiation of granulocytes from APL cells. Recently, Hou et al. reported that the hepatic RIG-I expression can predict the survival and IFN-α therapeutic response in hepatocellular carcinoma [15]. Liu et al. further showed that RIG-I regulated MMP9 to suppress the migration and invasion of hepatocellular carcinoma cells [16]. Chen et al. also found that RIG-I was abnormally expressed in EBV-induced nasopharyngeal carcinoma, and its higher expression level was significantly associated with a better survival of the patients [17]. However, as of now, the exact role of RIG-I in the oncogenesis and cancer progression of human gastric cancer still remains investigation.

Since the infection of gastric mucosa by *H. pylori* can result to gastric cancer, there is increasing evidence supporting that many PRRs family members are involved in the oncogenesis and cancer progression of gastric cancer [5]. For example, TLR2 activation could up-regulate TLR4 expression through MEK1/2-ERK1/2 pathway, which finally contributed to gastric inflammation, rapid cell proliferation and subsequent carcinogenesis [18]. And TLR4 induced ROS production to significantly increase cancer cell proliferation as well [19]. However, the role of RLRs, which consists of RIG-I, MDA-5, and LGP2, in human gastric cancer still remains elusive. Tatsuta et al. demonstrated that that the increased MDA-5 expression could be found in human gastric antral mucosa with *H. pylori* infection, and the increased MDA-5 level was significantly associated with atrophy and intestinal metaplasia [20]. Kutikhin et al. suggested further oncogenomic investigations focus on polymorphisms in RIG-I based on their analysis on the oncogenic potential of RIG-I and MDA-5 [21]. Liu et al. have shown that RIG-I suppressed cell migration and invasion abilities through MMP9 in hepatocellular carcinoma [16]. And RIG-I, serving as a tumor suppressor, augmented STAT1 activation in hepatocellular carcinoma through its CARDs competitively binding to STAT1 against the negative regulator SHP1. In acute myeloid leukemia, RIG-I modulated Src-mediated AKT activation to restrain leukemic stemness [6].

In this study, we found that the decreased RIG-I expression was significantly correlated with the advanced pathological stage and the poorer prognoses of gastric cancer patients. The COX model suggested that the RIG-I expression level and the TNM stage could be used as independent prognostic predictors for gastric cancer patients. The results of cellular functional experiments demonstrated that RIG-I served as a tumor suppressor in human gastric cancer to restrain the cancer cell migration, proliferation and invasion, as well as control the cell cycle progression. Furthermore, our microarray analysis showed that the differentially expressed gene profiles were mainly enriched in certain signal pathways such as PI3K/Akt, and in certain biological processes. Therefore, combining previous reports and our results, it was demonstrated that loss of RIG-I promoted cancer progression in many human cancers. However, some studies have also shown that RIG-I facilitated the therapy resistance and expansion of breast cancer. And under certain circumstances, it recognized non-coding RNAs and endogenous retroviruses and promoted T cell-independent B cell activation through IFN signaling activation. Therefore, the exact role of RIG-I in oncogenesis and development of gastric cancer still needs to be explored in further investigations.

## Conclusions

Our present findings demonstrated that the decreased RIG-I expression was significantly associated with poor prognosis, and such down-regulation could promote the cell invasion in human gastric cancer.

## Additional files

**Additional file 1.**  Gene profile after knockdown of RIG-I in AGS.

**Additional file 2.** Gene profile after knockdown of RIG-I in SGC-7901.

## Abbreviations

RIG-I: retinoic acid-induced protein I
PPRs: pattern recognition receptors
PAMPs: pathogen-associated molecular patterns
DAMPs: danger-associated molecular patterns
